# Reliability and Validity of a Point-of-Care Sural Nerve Conduction Device for Identification of Diabetic Neuropathy

**DOI:** 10.1371/journal.pone.0086515

**Published:** 2014-01-22

**Authors:** Justin A. Lee, Elise M. Halpern, Leif E. Lovblom, Emily Yeung, Vera Bril, Bruce A. Perkins

**Affiliations:** 1 Division of Endocrinology and Metabolism, Department of Medicine, University of Toronto, Ontario, Canada; 2 Division of Neurology, Department of Medicine, University of Toronto, Ontario, Canada; Medical University Innsbruck, Austria

## Abstract

**Background:**

Confirmation of diabetic sensorimotor polyneuropathy (DSP) relies on standard nerve conduction studies (NCS) performed in specialized clinics. We explored the utility of a point-of-care device (POCD) for DSP detection by nontechnical personnel and a validation of diagnostic thresholds with those observed in a normative database.

**Research Design and Methods:**

44 subjects with type 1 and type 2 diabetes underwent standard NCS (reference method). Two nontechnical examiners measured sural nerve amplitude potential (SNAP) and conduction velocity (SNCV) using the POCD. Reliability was determined by intraclass correlation coefficients (ICC [2], [1]). Validity was determined by Bland-Altman analysis and receiver operating characteristic curves.

**Results:**

The 44 subjects (50% female) with mean age 56±18 years had mean SNAP and SNCV of 8.0±8.6 µV and 41.5±8.2 m/s using standard NCS and 8.0±8.2 µV and 49.9±11.1 m/s using the POCD. Intrarater reproducibility ICC values were 0.97 for SNAP and 0.94 for SNCV while interrater reproducibility values were 0.83 and 0.79, respectively. Mean bias of the POCD was −0.1±3.6 µV for SNAP and +8.4±6.4 m/s for SNCV. A SNAP of ≤6 µV had 88% sensitivity and 94% specificity for identifying age-and height-standardized reference NCS values, while a SNCV of ≤48 m/s had 94% specificity and 82% sensitivity. Abnormality in one or more of these thresholds was associated with 95% sensitivity and 71% specificity for identification of DSP according to electrophysiological criteria.

**Conclusions:**

The POCD demonstrated excellent reliability and acceptable accuracy. Threshold values for DSP identification validated those of published POCD normative values. We emphasize the presence of measurement bias – particularly for SNCV – that requires adjustment of threshold values to reflect those of standard NCS.

## Introduction

Diabetic sensorimotor polyneuropathy (DSP) is the most common complication of diabetes affecting approximately 50% of individuals. [1], [2] It is thought that as many as half of individuals with DSP remain undiagnosed due to varying assessment practices among health care providers. [3], [4] Targeting this care gap may help to prevent progression of DSP to its clinical sequelae such as pain, loss of balance, foot ulceration, and limb amputation. [5], [6] These complications impose serious socioeconomic consequences as health care costs may double for those with DSP. [7], [8] Early detection of DSP is important for the prevention of disease progression and is critical for clinical research initiatives exploring primary and secondary interventions. [9], [10].

Detection of DSP requires intensive study in specialized neurology clinics using standard nerve conduction methods. These accepted gold-standard criteria rely on the presence of clinical signs and symptoms in addition to abnormal electrophysiological findings. [11], [12] Measurement of these electrophysiological parameters is time-consuming and expensive, and access to care is hindered by the limited number of clinics available to perform standard nerve conduction assessments in the face of the increasing prevalence of diabetes. [13], [14] There is a need to develop more rapid and more accessible methods of DSP identification that provide quantitative results that reasonably reflect those of standard nerve conduction studies (NCS). [15].

A novel point-of-care nerve conduction device (DPN-Check, Neurometrix Inc., Waltham, MA) has been developed that has the potential to serve as an acceptable proxy to standard NCS for screening and identification of DSP in clinical research and practice. An earlier alternate version of a point-of-care nerve conduction device was shown to have acceptable agreement with standard NCS parameters and accurately identified cases of DSP, but its adoption into clinical practice was limited by device complexity. [16], [17] The newest point-of-care device detects sural nerve amplitude potential (SNAP) and conduction velocity (SNCV) using the same principles as standard NCS, but is substantially more user-friendly and rapid, and can be used by examiners without prior training in standard NCS protocols. While standard NCS rely on a specialized technician to carefully place stimulating and recording electrodes anatomically over the sural nerve, the point-of-care device eliminates this need. Rather, the device uses a sensor pad to survey a broad area for signals from the sural nerve. However, some aspects of the device that make it practical may limit its accuracy. First, as opposed to standard NCS which stimulates the nerve antidromically, the point-of-care device uses orthodromic stimulation. Second, unlike standard NCS which depends on the expertise of a technician to iteratively stimulate the sural nerve until a valid response is detected, the point-of-care device may introduce error as it restimulates the nerve in an automated protocol. Validation of the reproducibility and accuracy of this device is needed in patients with diabetes as investigation of its technical performance has been limited to healthy populations [18].

DSP is a length-dependent and initially axonal neuropathy and therefore assessment of the sural nerve – the longest sensory nerve – has the greatest face validity as a single parameter for its identification. [19], [20], [21] SNAP and SNCV are quantitative measures that reflect the number of axons able to conduct impulses and the relative degree of myelination in the axons, respectively. [22] Therefore, sural nerve conduction parameters could be used to identify DSP both in clinical practice and research. [23] The purpose of this study was to evaluate the intra- and interrater reliability, accuracy in quantitative measurement, and diagnostic performance of the novel point-of-care nerve conduction device in patients with diabetes and a broad spectrum of nerve injury.

## Methods

### Ethics Statement

This protocol and consent procedures were conducted in accordance with the World Medical Association’s Helsinki Declaration and were approved by the Multidisciplinary Research Ethics Board of the Toronto General Hospital Research Institute. All participants provided written informed consent. We examined a cohort of 44 subjects, 16 with type 1 diabetes and 28 with type 2 diabetes. All subjects were accrued between September 1, 2012 and December 31, 2012 from two ongoing research studies at the Toronto General Hospital. Inclusion criteria were published previously. [24] In brief, subjects were excluded if they were under the age of 18 or presented with neuropathy not related to diabetes as determined by a detailed medical history, family history of neuropathy, history of toxin exposure, renal failure, or presence of abnormal serum or urine protein electrophoresis.

### Nerve Conduction Studies (Reference Method)

**Figure 1:**
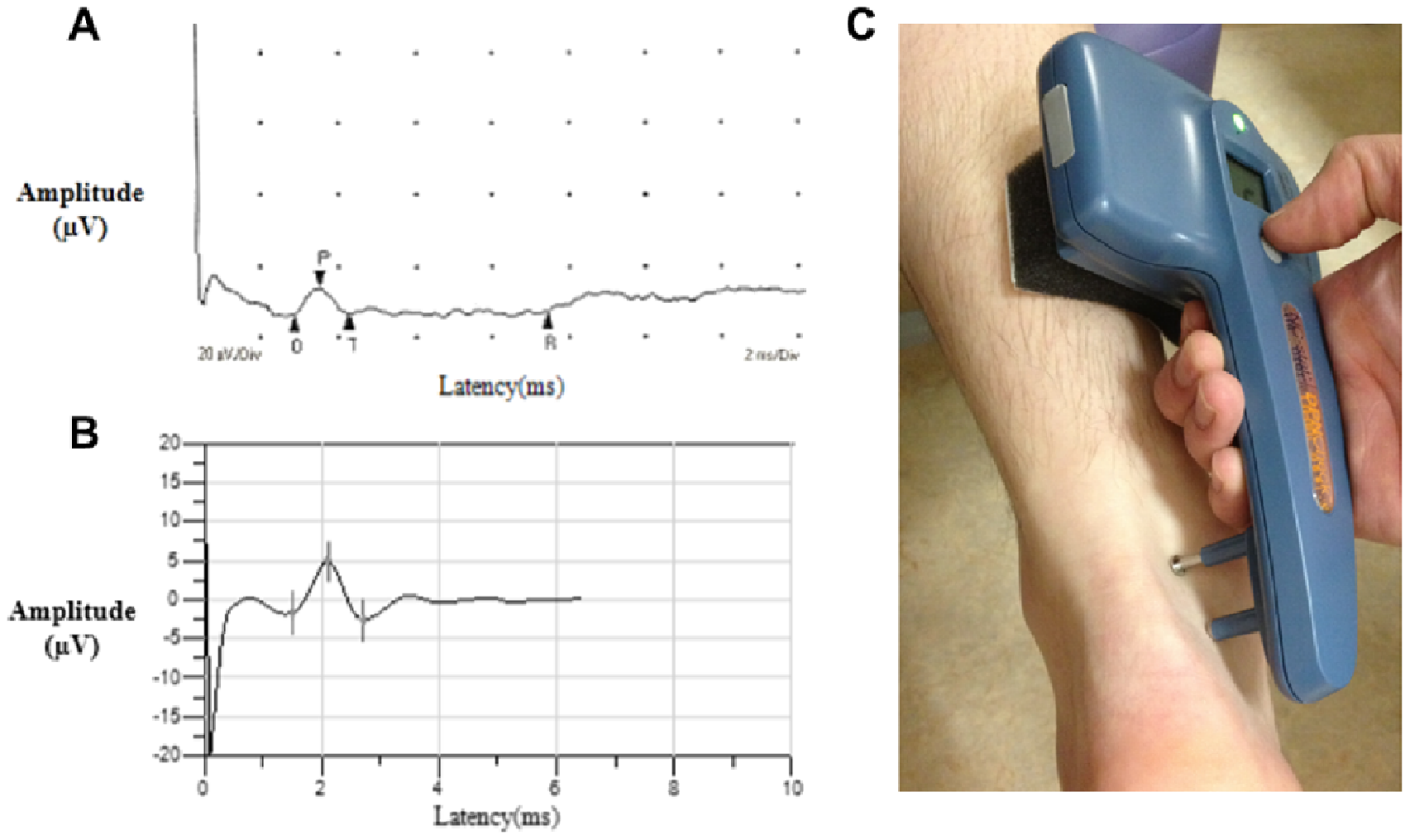
Sample nerve conduction recordings from standard NCS (A) and the point-of-care device (B) from a 60-year-old female with type 2 diabetes and an image of the point-of-care procedure (C). Panel A: Sample standard NCS recording. Sural nerve amplitude potential was 6.8 µV and conduction velocity was 48.3 m/s. Panel B: Sample recording from the point-of-care device. Sural nerve amplitude potential was 8 µV and conduction velocity was 56 m/s. Panel C: The device was placed on the lateral aspect of the leg and the sural nerve was stimulated and recorded by the electrical probes and biosensor, respectively.

Subjects underwent standard NCS on the left lower limb using the Sierra Wave instrument (Cadwell Laboratories, Kennewick, WA, USA). A sample NCS recording is shown in Figure 1 (Panel A). NCS were performed in accordance with the principles of the American Association for Neuromuscular and Electrodiagnostic Medicine. [25] In compliance with these standards, all sensory nerve conduction results were acquired following antidromic stimulation of the nerve. Stimulating probes were placed according to the discretion of the trained technician and limbs were maintained above 32°C. [11], [12], [13] The entire, comprehensive NCS procedure took 45–90 minutes to complete per patient. Peak-to-peak sural nerve amplitude potential and conduction velocity were measured at a fixed distance of 140 mm. Peroneal motor nerve amplitude potential, conduction velocity, and F wave were also recorded. In accordance with standard electrophysiological criteria, DSP was identified by at least one abnormal nerve conduction result in the sural sensory nerve and the peroneal motor nerve. [11], [12] Abnormal results pertaining to standard NCS were defined as being ≤1st percentile and ≥99th percentile in a healthy population after adjustment for age and height where applicable [23], [26].

### Point-of-Care Device (Test Method)

Subjects were examined bilaterally on the lower limb using the point-of-care nerve conduction device (DPN-Check, Neurometrix Inc., Waltham, MA). A sample recording from the point-of-care device and an image of the device are shown in Figure 1 (Panels B and C). Examinations were completed by two nontechnical personnel without prior training in standard NCS protocol, but who received one hour of training from a Neurometrix Inc. representative. The point-of-care device consisted of a single handheld unit that allowed for placement of a disposable biosensor at a fixed distance of 92.2 mm from the stimulating probes at the opposite end of the device. The biosensor covered a wide area to capture nerve conductions without the need for careful positioning by a specialized examiner. The device contained a built-in infrared thermometer located below the stimulating probes to detect ankle temperature. The device corrected for skin temperature, compensated for temperatures between 23 and 30°C, and prevented tests from beginning when ankle temperatures were below 23°C.

Located on the top of the device, opposite to the probes and biosensor, was a display screen, a single button, and an indicator light. The subject was instructed to assume one of two testing positions demonstrated by the examiner. The leg was prepared using a preparation pad that sterilized and buffered the testing area. The stimulating probes were coated in gel to promote conduction of the electrical impulse generated by the probes. To orient the device on the leg, the largest probe was placed on the lateral side of the ankle over the anatomical position of the sural nerve anterior to the Achilles tendon and posterior to and at the midline of the lateral malleolus – halfway between the two anatomical sites. With this probe in place the medial edge of the biosensor was placed on the lower calf in line with a proximal trajection of the Achilles tendon.

Once the device was in place the testing leg was selected on the display screen. The test was initiated when the button on the device was pressed once by the examiner. The nerve was then stimulated orthodromically. In an automated protocol, the sural nerve was stimulated 4–16 times within 10–20 seconds of the button being pressed. Stimulation number and duration depended on the strength of the sural nerve signal detected by the biosensor. Any results below 1.5 µV were automatically adjusted to zero by the device. The entire procedure was repeated twice bilaterally by two independent examiners. If a device error was observed the examiner was instructed to record the error and repeat the testing protocol. If a second error was obtained an additional test was permitted. A single recording took approximately two minutes to complete. The manufacturer has designated age- and height-adjusted thresholds for SNAP and SNCV as a measure of nerve conduction abnormality that are described in a normative database. [18] The order of the point-of-care device and standard NCS examinations was random.

### Statistical Analysis

Analyses were performed using SAS version 9.3 for Windows (SAS Institute, Cary, North Carolina). Clinical characteristics of the type 1 diabetes and type 2 diabetes groups were compared using the Student’s t-test (for normally distributed variables), the Wilcoxon rank-sum test (for non-normally distributed variables), or the χ^2^ test (for frequencies). A variable’s distribution was assessed using visual inspection and the Kolmogorov-Smirnov test for normality. Nerve conduction data was described using both mean±SD and median[IQR]. For all other variables, normally distributed variables were described using mean±SD while non-normally distributed variables were described using median[IQR]. For reliability (reproducibility) assessment, bilateral measurements were used. Intra- and interrater reliability was assessed using intraclass correlation coefficients (ICC). In particular, ICC class(2,1) was used as it reduces bias and considers the same raters to be a random subset of all possible raters in the population. [27], [28] Correlation coefficients >0.75 were considered to have excellent reliability. For the validity analysis, left-sided measures were used as standard NCS was performed unilaterally. Validity was determined in two ways. First, the statistical accuracy was determined quantitatively by a comparison of continuous values from the point-of-care device and standard NCS. Second, values from the point-of-care device and standard NCS were dichotomized into abnormal and normal results whereby their statistical agreement was assessed qualitatively. Statistical accuracy in the continuous variables, SNAP and SNCV, of the point-of-care device was compared to those produced by standard NCS using the method of Bland and Altman using the 85% confidence interval. [29] Diagnostic validity of the point-of-care device and resultant abnormal nerve conduction results were analyzed using receiver operating characteristic (ROC) curves. ROC curves were used to determine optimal threshold values in which the point-of-care device could distinguish between normal and abnormal readings for SNAP and SNCV. Using these thresholds a second ROC analysis compared normal and abnormal point-of-care device readings to DSP status as defined by standard NCS. Undetectable SNCV results for both nerve conduction methods were assigned a value of 30.4 m/s, the lowest value in the dataset. This applied to 9 observations for standard NCS and 3 observations for the point-of-care device.

## Results

Clinical characteristics of the 44 subjects with diabetes are depicted in Table 1. The cohort was comprised of 22(50%) females, mean age of 56±18 years, and mean diabetes duration of 18±14 years. Generally, the cohort was overweight with a mean body mass index of 27.7±5.0 kg/m^2^, hypertensive with a mean systolic blood pressure of 139±23 mmHg, and had target mean glycated hemoglobin A_1c_ (HbA1c) of 7.0±1.1%. DSP was present in 22(50%) subjects. The mean SNAP and SNCV measured by standard NCS were 8.0±8.6 µV and 41.5±8.2 m/s, respectively. The point-of-care nerve conduction device had a mean SNAP of 8.0±8.2 µV and SNCV of 49.9±11.1 m/s. In Table 1 we also present the clinical characteristics of subjects according to presence of type 1 or type 2 diabetes. The 28 subjects with type 2 diabetes were older, had shorter diabetes duration, and lower HbA1c than the 16 subjects with type 1 diabetes.

**Table 1:** Clinical characteristics of 44 subjects with type 1 and type 2 diabetes.

Characteristic	Total Cohort	Type 1 Diabetes	Type 2 Diabetes	P-value
N	44	16	28	–
Age (years)	56±18	45±18	62±15	0.001
Female sex, n(%)	22 (50)	9 (56)	13 (46)	0.53
Diabetes duration (years)	12.5 [7], [29]	29.5[16.5,38]	11 [5], [14]	0.001
Current/recent smokers, n(%)	5 (12)	1 (6)	4 (14)	0.61
Height (m)	1.70±0.11	1.67±0.11	1.72±0.10	0.13
Weight (kg)	80.4±15.7	77.6±14.3	82.1±16.5	0.38
BMI (kg/m^2^)	27.7±5.0	27.9±5.4	27.5±4.8	0.79
Systolic BP (mmHg)	139±23	138±26	139±22	0.89
Diastolic BP (mmHg)	76±13	73±9	77±14	0.40
HbA_1c_ (%)	7.0±1.2	7.7±0.9	6.7±1.2	0.01
Total cholesterol (mmol/L)	4.4±1.2	4.1±0.7	4.6±1.4	0.14
Triglycerides (mmol/L)	1.3±0.8	1.1±0.8	1.5±0.8	0.21
TCNS	7.5[1,11.5]	5[0,9.5]	9[3.5,12.5]	0.11
Presence of DSP, n(%)	22 (50)	7 (44)	15 (54)	0.51
Standard nerve conductions studies				
Sural nerve amplitude potential (µV)				
mean	8.0±8.6	7.3±7.3	8.3±9.4	0.67
median	4.7[2.3,12.8]	4.4[1.9,14.4]	5.5[2.3,12.3]	
Sural nerve conduction velocity (m/s)				
mean	41.5±8.2	41.2±8.1	41.7±8.5	0.83
median	42.4[32.7,46.7]	42.4[33.6,46.7]	42.4[32.7,47.5]	
Point-of-care device				
Sural nerve amplitude potential (µV)				
Mean	8.0±8.2	7.2±6.6	8.3±8.9	0.78
median	5.5 [3], [11]	4 [3], [13]	7 [3], [11]	
Sural nerve conduction velocity (m/s)				
mean	49.9±11.1	49.6±11.7	50.1±11.1	0.91
median	49.5[43.5,58]	48 [44,57]	50 [43,59]	

Data are mean±SD or median[IQR] unless otherwise stated. P-value indicates level of significance between type 1 and type 2 diabetes subjects. Differences were assessed using the χ^2^ test for categorical variables, the Student’s t-test for normally distributed variables, and the Wilcoxon rank-sum test for non-normally distributed variables.

BMI = body mass index; BP = blood pressure; HbA_1c_ = glycated haemoglobin A_1c_; TCNS = Toronto clinical neuropathy score; DSP = diabetic sensorimotor polyneuropathy.

As seen in Table 2, the point-of-care nerve conduction device demonstrated excellent intrarater reliability for both parameters in all subjects, with ICC values of 0.97 and 0.94 for SNAP and SNCV, respectively. Similarly, interrater reliability demonstrated excellent agreement for SNAP and SNCV with ICC values of 0.83 and 0.79, respectively. We did not observe substantial differences in intra- and inter-rater reproducibility between subjects with type 1 and type 2 diabetes.

**Table 2:** Intra- and interrater reliability of the sural nerve amplitude potential and conduction velocity using the point-of-care device for 44 subjects with type 1 and type 2 diabetes.

	Total cohort (n = 44)		Type 1 Diabetes (n = 16)		Type 2 Diabetes (n = 28)	
	Median (IQR)	ICC	Median (IQR)	ICC	Median (IQR)	ICC
Intra-rater reliability						
SNAP (µV)	6 (3–11)	0.97	5 (3–13)	0.97	5 (2–9)	0.97
SNCV (m/s)	50 (43–58)	0.94	54 (45–58.5)	0.96	48 (36–54)	0.94
Inter-rater reliability						
SNAP (µV)	5 (2–10)	0.83	4 (2–6)	0.74	3 (2–8)	0.86
SNCV (m/s)	50 (41–58)	0.79	53 (44–58)	0.68	46 (33–54)	0.85

IQR = Interquartile range; ICC = Interclass correlation coefficients class(2,1); SNAP = sural nerve amplitude potential; SNCV = sural nerve conduction velocity.

**Figure 2:**
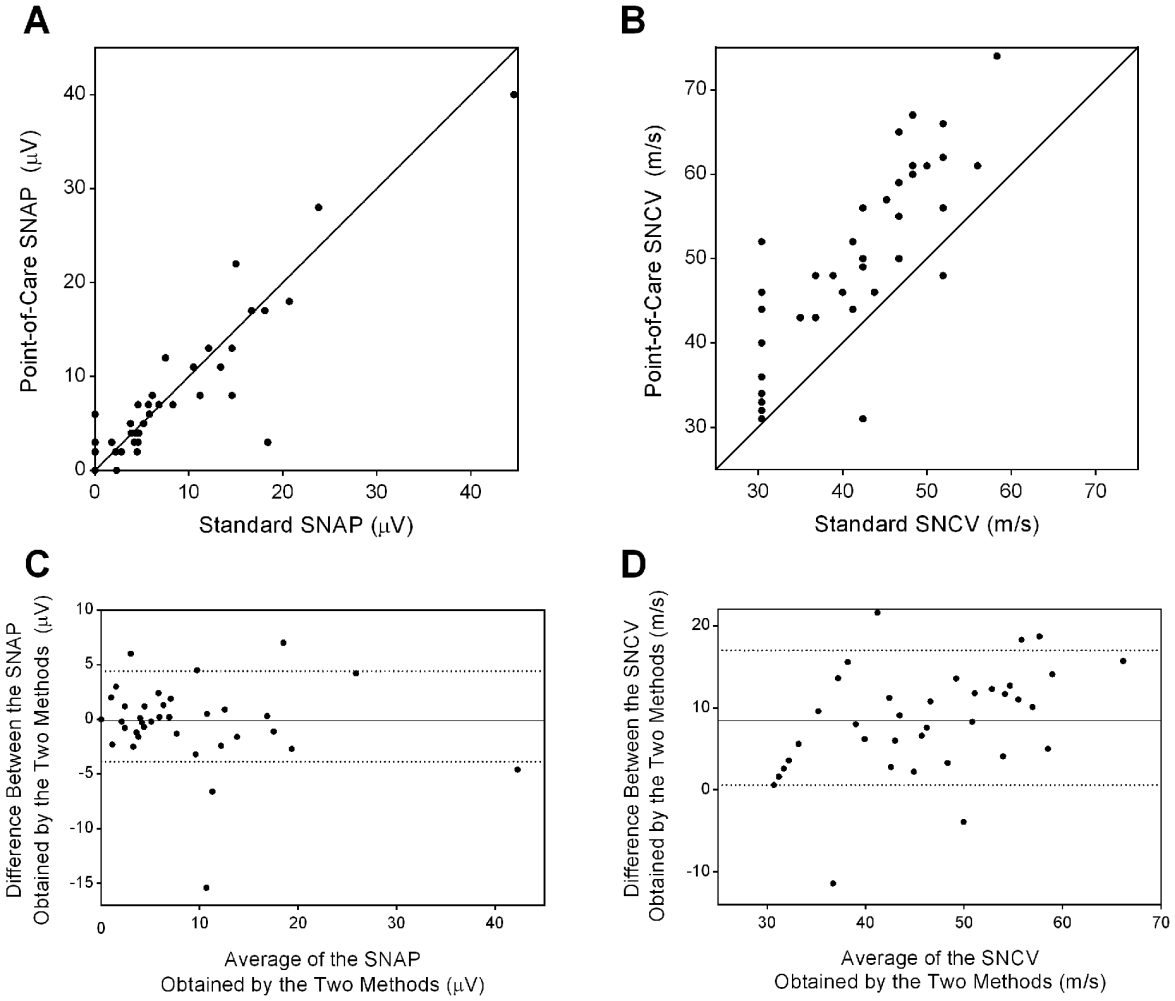
Scatterplots (A,B) and Bland-Altman plots (C,D) for comparison of the point-of-care nerve conduction method versus standard NCS for SNAP or SNCV. Panels A and B: Scatterplot of SNAP (A) and SNCV (B) showing the line of unity (diagonal solid line) between the two methods. Panels C and D: The Bland-Altman plots demonstrating the mean difference (depicted by the solid line) between SNAP (C) or SNCV (D) obtained by the two methods. Points above or below zero on the y-axis represent over- and underestimation by the point-of-care device, respectively. The dotted lines represent the upper and lower limits of the 85% confidence interval. Unrecordable SNCV results for both nerve conduction methods were assigned a value of 30.4 m/s, representing the lowest value in the dataset. Such data handling was applied to 9 values for standard NCS and 3 values for the point-of-care device.

The quantitative accuracy of the point-of-care nerve conduction device for the continuous variables SNAP and SNCV is depicted graphically using scatterplots and Bland-Altman plots in Figure 2. This analysis demonstrated strong agreement between SNAP as measured by the point-of-care device and standard NCS with a mean difference of −0.1±3.6 µV and a median difference of +0.1 µV [85% confidence interval, −3.9 to +4.4 µV]. Agreement between SNCV measured by the point-of-care device and standard NCS shows a consistent overestimation by the point-of-care device by a mean of +8.4±6.4 m/s and median of +9.3 m/s [85% confidence interval, +0.6 to +17 m/s].

The presence of measurement bias led to an exploration of unique threshold values that could differentiate between normal and abnormal SNAP and SNCV. The threshold values that maximized sensitivity and specificity for the identification of abnormal values determined by ROC curve analysis were ≤6 µV and ≤48 m/s for SNAP and SNCV, respectively. For identification of abnormal SNAP, the ROC curve had an area under the curve (AUC) of 0.95 and the threshold value had a sensitivity of 88% and a specificity of 94%. For identification of abnormal SNCV, the ROC curve had an AUC of 0.92 and the threshold value had a sensitivity of 94% and a specificity of 82%.

**Figure 3:**
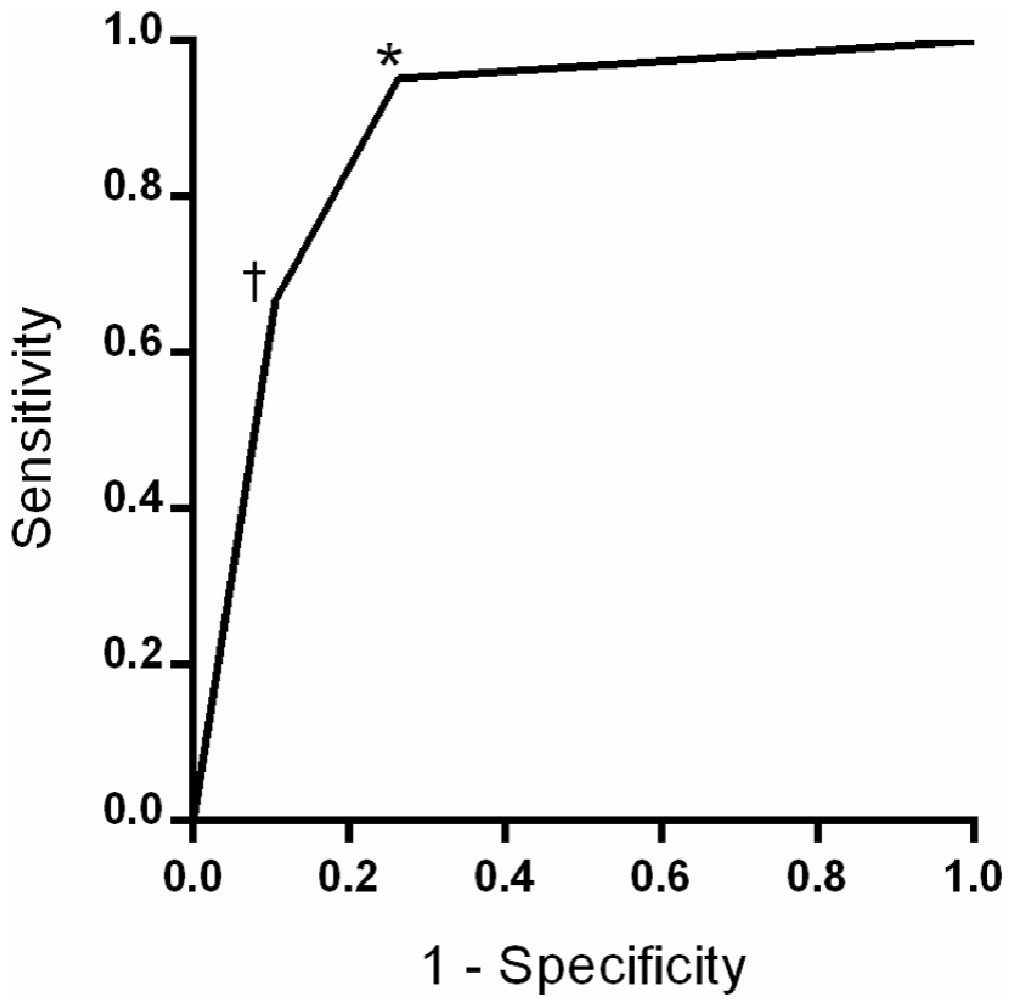
ROC curve showing the diagnostic validity of the point-of-care device for the identification of DSP as defined by electrophysiological criteria from standard NCS. An optimal threshold of one abnormality in SNAP or SNCV (*) had a sensitivity and specificity of 95% and 71%, respectively. An optimal threshold of abnormalities in both parameters (†) had a sensitivity and specificity of 67% and 89%, respectively.

The validity of the point-of-care nerve conduction device in the identification of DSP was also assessed according to whether one abnormal SNAP or SNCV was sufficient for the identification of DSP. As shown in Figure 3, the AUC was 0.88 and one or more abnormality in SNAP or SNCV had a sensitivity of 95% and specificity of 71%.

## Discussion

We examined a cohort of type 1 and 2 diabetes subjects with a broad spectrum of nerve injury, of which 22(50%) had DSP, to assess the performance of a novel point-of-care nerve conduction device in the identification of DSP. The device demonstrated excellent intrarater and interrater reproducibility, acceptable accuracy, and good diagnostic validity for the identification of DSP defined electrophysiologically. The level of reproducibility of the point-of-care device appeared to be comparable to reproducibility measures from standard NCS. [30] However, while quantitative accuracy was excellent for SNAP, we observe a systematic overestimation of SNCV by the point-of-care device. From this measurement bias, we determined that adjustment of threshold values is required – either automatically within the programming of the device or in the published normative values for the device - to adequately reflect those of standard NCS.

On the quantitative scale, we demonstrated that the point-of-care nerve conduction device was accurate for SNAP with minimal bias as compared to standard NCS. Though we did not observe substantial bias, there are two factors that could lead to underestimation by the point-of-care device. First, SNAPs that are less than 1.5 µV are automatically adjusted to a level of zero by the device protocol. Second, the point-of-care device stimulates the nerve orthodromically rather than antidromically which would typically result in a lower sensory amplitude potential. [31], [32] The device, however, is configured such that conduction distance, electrode spacing, and filter settings maximize amplitude to improve the signal to noise ratio. In this study, we report that impact of zeroed values and orthodromic stimulation by the point-of-care device is approximately balanced by the factors that maximize amplitude potentials.

In opposition to our findings for SNAP, however, the quantitative accuracy of SNCV was found to be substantially impaired. We observed a systematic overestimation of SNCV by the point-of-care device by a mean of +8.0 m/s which we could not explain by the analytical protocol or the nature of orthodromic stimulation as we could hypothesize for the minimal bias associated with measurement of SNAP. [31], [32] Instead, this systematic overestimation may be explained by two technical factors of the device that differ from standard NCS. First, the measurement of latency on the point-of-care device begins at the completion of the pulse on the stimulating electrode rather than at the initiation of the pulse as is customary for standard NCS. Second, the temperature correction algorithm differs from that of standard NCS. These two factors likely account for the majority of the +8.0 m/s bias, and have been addressed in subsequent versions of the point-of-care device software. [18] However, it is also possible that efficiencies in signal detection by the broad biosensor pad, as compared to manual positioning of standard NCS electrodes, may have contributed to this bias.

In spite of the systematic overestimation observed with SNCV, the device was able to qualitatively identify abnormality in standard NCS parameters and the presence or absence of DSP extremely well. As determined by ROC curve analysis, we found optimal thresholds of ≤6 µV and ≤48 m/s had excellent operating characteristics for the identification of age- and height-adjusted abnormality in the SNAP and SNCV measured by standard NCS. Although the magnitude of the SNAP threshold was in agreement with our laboratory’s standard NCS lower limit of amplitude potential, the value for SNCV exceeded our laboratory’s value by approximately 6 m/s to 8 m/s, depending on subject’s age and height. [23] However, these threshold values are consistent with established lower limits of the point-of-care device’s nerve conduction values found in an independent study. [18] In addition, we determined that a simple protocol in which abnormality in point-of-care SNAP, SNCV, or both was associated with high sensitivity (95%) and acceptable specificity (71%) for identification of DSP. These operating characteristics are consistent with the view that this device could be used to identify DSP with acceptable levels of accuracy in clinical research settings.

The measurement bias that we observed for SNCV both in our study and in the normative database [18] needs to be reconciled in order for the point-of-care device to have maximal utility. As the finding appears to be systematic in that we see this bias across the range of SNCV values in this analysis, and in that it is consistent between our study and the published normative database, [18] the bias can be reconciled by simple arithmetic adjustment of the point-of-care nerve conduction device results. Such adjustment for bias could be accounted for by one of two strategies. First, a separate threshold value for abnormal SNCV could be reported for the point-of-care device that differs from that of standard NCS. Alternatively, an adjustment algorithm could be programmed by the manufacturer in the device’s internal software so that adjusted values are reported directly to the examiner. Despite an observed overestimation of SNCV by the point-of-care device, its systematic nature permits solutions that do not limit its applicability.

Abnormal nerve conduction results are considered to be the gold standard objective test for confirmation of DSP. [11], [12] Current clinical practice guidelines recommend the use of simple physical examination maneuvers to screen for the presence of DSP or its future risk. [21], [33], [34] Although capable of identifying DSP with reasonable accuracy, these physical tests are subjective, rely on patient feedback, and may not have sufficient reproducibility. [35], [36] The point-of-care device is an appealing method for DSP identification as it is an objective quantitative measure that corresponds well with accepted nerve conduction studies.

Our findings suggest this point-of-care device could provide valid nerve conduction measures that can be used as a confirmatory test for DSP with high reproducibility, acceptable accuracy, and excellent validity. However, the study has limitations. First, the study group was relatively small and examined at a single investigational site and therefore may not be generalizable to a broader population. The study, however, was intended as a targeted validation of a much larger normative database in a diabetes population. [18] Second, the point-of-care device evaluated the performance of a single nerve despite the fact that DSP affects peripheral nerves diffusely. Third, we did not assess the impact of calf size to determine performance variability. Fourth, generalizability to specific clinical and research settings requires an estimation of cost-effectiveness – including the impact of false positive and false negative results – which was not examined in this study. Finally, we did not evaluate the point-of-care tool as a component of a clinical management protocol in which other, more simplified physical examination tests, were used to stratify which patients receive testing with the point-of-care nerve conduction device.

Though more work is needed, this study served to validate the existence of a point-of-care sural nerve conduction device in patients with diabetes for the identification of DSP with excellent reproducibility and sufficient validity for use in clinical research.
