# Correction: Reliability and Validity of a Point-of-Care Sural Nerve Conduction Device for Identification of Diabetic Neuropathy

**DOI:** 10.1371/journal.pone.0106981

**Published:** 2014-08-22

**Authors:** The PLOS ONE Staff

There is an error in the penultimate sentence of the Results section of the Abstract. The correct sentence is: A SNAP of ≤6 µV had 88% sensitivity and 94% specificity for identifying age-and height-standardized reference NCS values, while a SNCV of ≤48 m/s had 94% sensitivity and 82% specificity.”
